# Comparative efficacy of prophylactic protocols in reducing perioperative nausea and vomiting during video-assisted thoracoscopic radical resection of lung cancer

**DOI:** 10.1038/s41598-024-59687-z

**Published:** 2024-04-29

**Authors:** Weiguang Zhang, Zhimin Shen, Junfei Jiang, Shujing Zhu, Peipei Zhang, Sui Chen, Mingqiang Kang

**Affiliations:** 1 Department of Thoracic Surgery, Fujian Medical University Union Hospital, Fuzhou, China; 2 Key Laboratory of Ministry of Education for Gastrointestinal Cancer, Fujian Medical University, Fuzhou, China; 3 Fujian Key Laboratory of Tumor Microbiology, Fujian Medical University, Fuzhou, China; 4 The School of Basic Medical Sciences, Fujian Medical University, Fuzhou, China; 5 Clinical Research Center for Thoracic Tumors of Fujian Province, Fuzhou, China

**Keywords:** Quality of life, Cancer, Lung cancer

## Abstract

Lung cancer, a global mortality leader, often necessitates Video-Assisted Thoracoscopic (VATS) surgery. However, post-operative nausea and vomiting (PONV) is common, highlighting a need for effective management and prevention strategies in this context. A retrospective case–control study at Fujian Medical University Union Hospital evaluated patients undergoing VATS radical resection of lung cancer between May and September 2022. Patients were categorized based on PONV prevention methods, and data encompassing demographics, surgical history, and postoperative adverse events s were analyzed to assess the association between prophylactic protocols and PONV incidence. The Netupitant and Palonosetron Hydrochloride (NEPA) group showed a significant reduction in PONV occurrences post-surgery compared to Ondansetron (ONDA) and Control groups, emphasizing NEPA’s efficacy in alleviating PONV symptoms (P < 0.05). Furthermore, following VATS radical resection of lung cancer, NEPA markedly reduced the intensity of PONV symptoms in patients. Both univariate and multivariate logistic analyses corroborated that NEPA independently reduces PONV risk, with its protective effect also apparent in susceptible populations like females and non-smokers. NEPA utilization markedly reduced both the incidence and severity of PONV in patients undergoing VATS radical resection of lung cancer, serving as an independent protective factor in mitigating PONV risk post-surgery.

## Introduction

Lung cancer is a globally prevalent malignancy associated with a significant mortality rate. In China, it exhibits the highest mortality rate among all types of malignant tumors^1^. Among men, lung cancer demonstrates a notably high incidence and mortality rate. Among women, it ranks as the second most common cancer, following breast cancer, yet it maintains the highest mortality rate^2^. Despite remarkable progress in adjuvant therapies, including chemotherapy, radiotherapy, and targeted therapy, the survival rate of lung cancer has shown limited improvement in recent years. According to the National Comprehensive Cancer Network guidelines, thoracoscopic surgery is recommended as the preferred procedure for radical resection of lung cancer^3^. The term “radical resection” of lung cancer delineates an extensive surgical strategy that seeks to excise the cancerous tissue entirely, encompassing a margin of surrounding healthy tissue as well as any affected lymph nodes. This approach underscores the imperative of achieving a thorough removal to minimize the risk of recurrence and to enhance the prognosis of the patient^4^. Over the years, thoracoscopic radical resection for lung cancer has gained widespread acceptance and has become a standardized approach in the management of this disease^5^. In recent years, there has been a significant surge in the recognition and implementation of the rapid recovery concept within the field of surgery. During the perioperative period of thoracic surgery, the routine adoption of postoperative multimodal analgesia has become widespread. This approach combines various analgesic medications and techniques to effectively alleviate pain and enhance patient comfort. However, it is important to acknowledge that the extensive use of anesthetic and analgesic drugs has been linked to a notable incidence of post-operative nausea and vomiting (PONV) among patients.

PONV is a frequently encountered complication following anesthesia and surgery, with an incidence ranging from 20 to 30%^6^. In patients undergoing lobectomy for lung cancer under general anesthesia, the incidence of PONV was reported to be 29.6%^7^. PONV can give rise to various adverse events, such as aspiration, wound dehiscence, bleeding, dehydration, electrolyte imbalance, and the inability to initiate oral medication^8,9^. Additionally, PONV can contribute to delayed recovery and pose life-threatening complications, including hospital-acquired pneumonia and thromboembolic events^10^. The management of PONV presents challenges due to its multifaceted etiology, encompassing patient characteristics, surgical factors, and anesthetic techniques, compounded by the variable response to prophylactic antiemetics^11,12^. Furthermore, the inconsistency in clinical practice guidelines exacerbates these challenges^13^. Consensus guidelines for managing PONV highlight several risk factors associated with its occurrence. These include being female, younger age, undergoing high-risk surgery, exposure to volatile anesthetics, a history of PONV or motion sickness, non-smoking status, administration of post-operative opioids, and prolonged anesthesia duration^14^. Studies have indicated that female patients and non-smokers undergoing lobectomy are more likely to experience a higher incidence of PONV^7^. Furthermore, increased doses of fentanyl per kilogram per hour have been identified as an independent risk factor for PONV during thoracoscopic lung wedge resection^15^. Given the high prevalence, associated risks, and complex management of PONV, it is crucial to conduct ongoing research to optimize prevention and treatment strategies.

Ondansetron (ONDA) is a selective antagonist of 5-hydroxytryptamine (5-HT3) receptors, which are involved in serotonin-mediated nausea and vomiting. By inhibiting these receptors, ondansetron effectively reduces the onset of these symptoms^16^. ONDA and other 5-HT3 receptor antagonists, often used in combination with metoclopramide for PONV treatment, demonstrate a 21% incidence of PONV and are well tolerated with a proven safety profile in clinical settings^17,18^. Netupitant and Palonosetron Hydrochloride (NEPA), comprising netupitant and palonosetron, effectively prevents and manages nausea and vomiting^19^. Netupitant acts as a neurokinin-1 (NK-1) receptor antagonist, blocking substance P in the vomiting reflex^20,21^. Palonosetron induces a prolonged inhibition of 5-HT3 receptor function and additionally disrupts the interaction between 5-HT3 and NK-1 receptors^22^. Moreover, research indicates that the concomitant administration of palonosetron and netupitant synergistically amplifies the suppression of the substance P-mediated response, surpassing the efficacy observed with either antagonist individually^23^. Based on the information above, various medications exist for preventing and managing PONV. Nevertheless, there is a lack of comparative studies assessing different antiemetic regimens’ impact on PONV remission in patients undergoing VATS radical resection of lung cancer. This research gap hampers the identification of optimal prevention strategies for PONV in this surgical context.

## Methods

### Patient selection and experimental design

This retrospective case–control study was conducted at the Department of Thoracic Surgery, Fujian Medical University Union Hospital. The study included patients who underwent video-assisted thoracoscopic surgery (VATS) radical resection of lung cancer between May 2022 and September 2022, and all procedures were performed by the same surgical team. The inclusion criteria were as follows: (1) Pathologically confirmed diagnosis of non-small cell lung cancer. (2) No chemotherapy or radiotherapy prior to surgery. (3) Age over 18 years. (4) Surgery not expected to be converted to open thoracic surgery. The following exclusion criteria were applied to ensure the accuracy and reliability of the study results: (1) Patients with serious heart disease, liver and kidney dysfunction, or other major internal medical diseases. (2) Patients with a history of recurrent vomiting or chronic use of antiemetic drugs in the past 6 months. (3) Patients allergic to the components of the study drug. (4) Women known to be pregnant or breastfeeding before surgery. (5) Patients with uncertain occurrence and severity of complications such as nausea and vomiting within 3 days after surgery during the data collection period. (6) Surgeries performed under the assistance of the da Vinci robot. The study protocol received approval from the Institutional Review Board of the Fujian Medical University Union Hospital (2023KY183), and the requirement for informed consent was waived by the review board due to the retrospective nature of the study. The research was carried out in compliance with the appropriate guidelines and regulations, adhering to the ethical principles for medical research involving human subjects as outlined in the Declaration of Helsinki.

The methodology for preventing PONV in this study was derived from a retrospective analysis of the treatment regimens recorded in the patients’ medical records. Patients were stratified into three distinct groups based on the prophylactic treatment received: the NEPA group, comprising 72 patients; the ONDA group, consisting of 70 patients; and the Control group, which included 66 patients. The grouping was based on the specific postoperative treatment regimen used to PONV. In the NEPA group, all patients consumed a small amount of warm water orally within 6 h after surgery and took 1 NEPA capsule. Each capsule contains 0.3 g of Netupitant and 0.5 mg of Palonosetron Hydrochloride. In the ONDA group, patients received a prophylactic intravenous injection of ONDA (0.25 mg) 0.5 h after surgery. Patients in the Control group did not receive any specific interventions for the prevention of PONV.

### Outcome measurement and data collection

Patient data, including gender, age, tobacco use, surgical history, BMI, history of hypertension, history of diabetes mellitus, operative time, and additional pain killer usage and postoperative hospitalization stay were collected from the patient medical record system of Union Hospital, Fujian Medical University. The evaluation of postoperative major adverse events in patients was systematically conducted using a bifurcated approach, incorporating both questionnaire surveys and telephone interviews. This methodology was applied based on the duration of postoperative hospitalization. For patients whose postoperative hospital stay exceeded 72 h, the assessment was exclusively conducted through electronic questionnaires administered at the time of their discharge. In contrast, for patients with a postoperative hospitalization period of less than 72 h, the evaluation of major adverse events entailed a dual approach: an electronic questionnaire provided at discharge, supplemented by a follow-up telephone interview. Furthermore, in cases where uncertainties or ambiguities arose regarding the patient's condition or responses, a dedicated staff member was available to conduct a thorough reassessment via a return telephone call. It is important to note that all patients involved in this study were proficient in reading Chinese and did not exhibit any clinically significant cognitive impairments, thereby ensuring the accuracy and reliability of the assessment process.

The severity of the following major adverse events was evaluated using the specified criteria:

- Nausea: 0 (none), 1 (mild: 1–2 occurrences), 2 (moderate: 3–5 occurrences), 3 (severe: 6 occurrences or more).
- Vomiting: 0 (none), 1 (mild: 1–2 occurrences), 2 (moderate: 3–4 occurrences), 3 (severe: 5 occurrences or more).
- Pain intensity: Pain levels were assessed using the Visual Analogue Scale (VAS) ranging from 0 to 10, with 0 representing no pain and 10 indicating the most severe pain. The pain scores were classified as follows: 0 (none: 0 points), 1 (mild: 1–3 points), 2 (moderate: 4–6 points), 3 (severe: 7–10 points).
- Dizziness: 0 (none), 1 (mild: very little dizziness), 2 (moderate: more frequent dizziness), 3 (severe: very frequent dizziness).

Additionally, data on postoperative vital signs, adverse events, and length of hospital stay were meticulously documented.

### Surgical, anesthesia, and analgesia methods

All patients included in this analysis were subjected to three-port VATS radical excision of lung cancer at our facility. The surgical approach entailed three distinct incisions: a primary operating incision measuring 3 cm at the fourth interspace, directly above the hilum; a secondary operating incision of 1.5 cm located at the ninth interspace along the posterior axillary line and a camera port incision of 0.5 cm at the seventh interspace on the midaxillary line. The primary and secondary incisions facilitated the insertion of surgical instruments into the thoracic cavity by the lead surgeon and the first assistant, while the camera port incision provided access for the thoracoscopic visualization equipment. To mitigate trauma to the patient’s musculature and skin at the incision sites, all three were safeguarded with incision protectors. Subsequent to the lesion’s excision, immediate intraoperative frozen section analysis was employed to verify the absence of cancerous cells at the resection margins, thereby confirming the thoroughness of the malignancy’s removal.

The anesthesia protocol for the patient was categorized into two phases: induction and maintenance. For the induction of anesthesia, a combination of intravenous agents was utilized, comprising propofol at a dosage of 1.0–2.5 mg/kg, sufentanil at 0.4–1.0 µg/kg, and rocuronium bromide at 0.6 mg/kg to facilitate rapid onset and optimal conditions for intubation. Maintenance of anesthesia was managed through a balanced technique, incorporating sevoflurane at a concentration of 1.5–2.5%, supplemented with continuous infusions of propofol at 1.5–4.5 mg/kg and remifentanil at a rate of 0.05–0.3 µg/kg/min. This approach aimed to ensure stable anesthesia depth, pain control, and muscle relaxation throughout the procedure.

During the postoperative period, patient pain management was systematically administered via an analgesic infusion pump. The pump dispensed a solution containing 4 mg of buprenorphine and 100 µg of sufentanil, diluted in 150 ml of normal saline (NS). This regimen was delivered at a basal rate of 4 ml/hour to maintain steady and controlled analgesia. Additionally, for enhanced pain relief, certain patients received an intramuscular injection of Bucinnazine Hydrochloride, administered at a dosage of 100 mg. Participants in this study were limited to receiving a maximum of one injection. This adjunct treatment approach was customized according to the specific needs and pain evaluations of each patient.

### Measures of reducing bias and confounders

To enhance the rigor of our retrospective study and mitigate potential biases, we implemented several measures to ensure the accuracy and reliability of our results. In the data collection phase, we established a comprehensive data extraction template that included patients' baseline characteristics, the occurrence and severity of PONV, and other relevant variables. This template was designed to standardize the collection of information across all participants. To minimize errors in data entry, all data were independently extracted and cross-checked by two researchers.

In the statistical analysis phase, we employed multivariate logistic regression to assess the independent effect of different treatment on preventing PONV, while adjusting for potential confounders such as age, gender, smoking history, surgical duration, and use of additional analgesics. This approach allowed us to isolate the impact of NEPA and ONDA on PONV risk from the influence of these confounding factors, providing a more accurate estimate of the treatment effect.

Additionally, to further control for the effects of smoking and gender, we conducted subgroup analyses using logistic regression in non-smokers and female patients, respectively. These analyses aimed to clarify the therapeutic effects of different treatment options on the risk of PONV occurrence in these specific subpopulations.

### Statistical analysis

Continuous variables with a non-normal distribution are reported as median (interquartile range), while continuous variables with a normal distribution are reported as mean (standard deviation). Categorical variables are presented as numbers (percentages). Independent samples t-test was used to assess the significance of normally distributed continuous variables, whereas the Mann–Whitney U-test was employed for abnormally distributed variables when comparing two groups. Chi-square test or Fisher's exact test was utilized to compare the frequencies of categorical variables. A univariate logistic regression analysis was conducted to examine the association between the use of NEPA and ONDA medications, as well as other clinical variables, with the incidence of PONV. Factors that showed statistically significant differences (P < 0.05) in this initial analysis were then included in a multivariate logistic regression analysis. This further analysis aimed to identify the independent influences of each variable on the occurrence of PONV. Statistical analysis was performed using R software (version 4.0.4), with p-values < 0.05 considered statistically significant.

## Results

### Participant flow and baseline characteristics: present the number of patients in each group and their demographic details

All the cases included in this study underwent radical resection of lung cancer using general anesthesia thoracoscopy. The total number of cases was 208, with 72 cases in the NEPA group, 70 cases in the ONDA group, and 66 cases in the Control group. No intermediate open-heart or palliative resections were performed, and there were no significant perioperative adverse events in any of the cases. Apart from the length of postoperative hospitalization stay, most clinical and demographic characteristics, including gender, age, tobacco use, surgical history, BMI, history of hypertension, history of diabetes mellitus, operative time, and additional pain killer, were well-balanced between the three groups (p > 0.05). It is noteworthy that the length of postoperative hospitalization stay for patients in the NEPA group was significantly shorter compared to those in the ONDA combo Control group (p = 0.00725). Table 1 provides a summary of the comparison of clinical baseline characteristics among the NEPA, ONDA, and Control groups.

**Table 1:** Comparative baseline clinical characteristics of the evaluated groups.

	NEPA (n = 72)	ONDA (n = 70)	Control (n = 66)	Total (n = 208)	P value
Gender					
Female	43 (59.72%)	47 (67.14%)	42 (63.64%)	132 (63.46%)	0.65568
Male	29 (40.28%)	23 (32.86%)	24 (36.36%)	76 (36.54%)	
Age					
Mean (SD)	56.9 (11)	54.1 (10.4)	55.3 (11)	55.5 (10.8)	0.295
Median [min, max]	58.5 [27,79]	56 [33,77]	53.5 [33,76]	56 [27,79]	
BMI					
Mean (SD)	22.1 (2.6)	22.6 (2.8)	23.1 (2.7)	22.6 (2.7)	0.2948
Median [min, max]	22.5 [16.6,27.5]	22.4 [14.2,28.6]	23 [18.6,31.1]	22.6 [14.2,31.1]	
Smoke					
No	52 (72.22%)	59 (84.29%)	50 (75.76%)	161 (77.40%)	0.21193
Yes	20 (27.78%)	11 (15.71%)	16 (24.24%)	47 (22.60%)	
Surgical history					
No	40 (55.56%)	45 (64.29%)	32 (48.48%)	117 (56.25%)	0.17658
Yes	32 (44.44%)	25 (35.71%)	34 (51.52%)	91 (43.75%)	
Hypertension					
No	51 (70.83%)	60 (85.71%)	50 (75.76%)	161 (77.40%)	0.0981
Yes	21 (29.17%)	10 (14.29%)	16 (24.24%)	47 (22.60%)	
Diabetes					
No	65 (90.28%)	63 (90.00%)	61 (92.42%)	189 (90.87%)	0.86661
Yes	7 (9.72%)	7 (10.00%)	5 (7.58%)	19 (9.13%)	
Operative time					
Mean (SD)	96.5 (26.7)	107.4 (33.1)	96.9 (35.5)	100.3 (32.1)	0.0745
Median [min, max]	97 [35,166]	108 [25,175]	95 [35,193]	100 [25,193]	
Additional pain killer					
No	63 (87.50%)	54 (77.14%)	51 (77.27%)	168 (80.77%)	0.20068
Yes	9 (12.50%)	16 (22.86%)	15 (22.73%)	40 (19.23%)	
Postoperative hospitalization stay					
Mean (SD)	4.2 (1.5)	5.1 (2.1)	5 (2.3)	4.7 (2.1)	0.00725
Median [min, max]	4 [2, 9]	5 [2, 15]	4.5 [2, 13]	4 [2, 15]	

### Comparison of major adverse event rates following VATS radical resection of lung cancer

**Figure 1:**
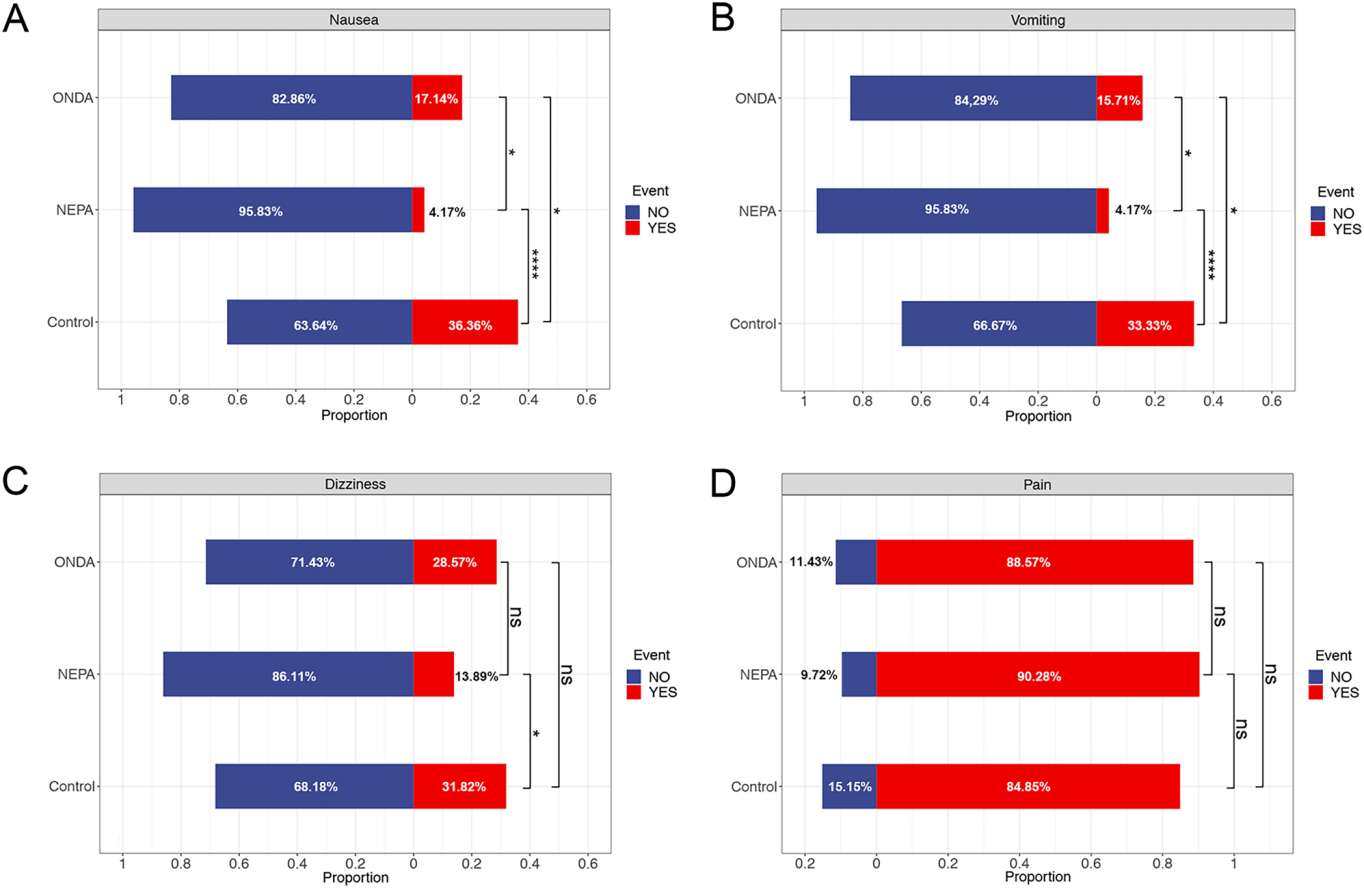
Comparison of postoperative major adverse events following VATS radical resection of lung cancer. The stacked bar graphs depict the incidences of four adverse events—nausea (**A**), vomiting (**B**), dizziness (**C**), and pain (**D**)—in the NEPA, ONDA, and Control groups over the first three postoperative days. The red bars (Event YES) indicate the percentage of patients who experienced each event, while the blue bars (Event NO) show the proportion of patients without these events. Percentages are noted within each bar. Significance levels are denoted as follows: *P < 0.05, **P < 0.01, ***P < 0.001, ****P < 0.0001.

In the NEPA group, after treatment with NEPA, the incidence of nausea and vomiting was 4.17% and 4.17%, respectively. In the ONDA group, which received ONDA, the incidence of nausea and vomiting was 17.14% and 15.71%, respectively. The Control group, without any pharmacological intervention, had an incidence of 36.36% for nausea and 33.33% for vomiting (Fig. 1A,B, Table S1, S2, S3).

The findings of this study reveal a noteworthy reduction in the occurrence of nausea and vomiting following pharmacological treatment in both the NEPA and ONDA groups, demonstrating a significant improvement (P < 0.05). Particularly, the use of NEPA exhibited a significantly superior reduction in PONV compared to ONDA (P < 0.05). In subgroup analyses focusing on women with heightened risk factors for PONV and in nonsmoking cohorts, NEPA consistently demonstrated superior efficacy over ONDA in mitigating nausea symptoms (Tables S4 and S5) In relation to dizziness, the incidences were 13.89%, 28.75%, and 31.82% in the NEPA, ONDA, and Control groups, respectively (Fig. 1C). The difference between the NEPA and ONDA groups was statistically significant (P < 0.05), highlighting the potential of NEPA to mitigate dizziness in patients undergoing VATS radical resection of lung cancer. However, there were no significant differences observed among the three groups regarding postoperative pain occurrence (p > 0.05, Fig. 1D). Detailed information regarding the incidence of major side effects following VATS radical resection of lung cancer and their comparative analysis can be found in Tables S1, S2 and S3.

Furthermore, we constructed a line graph to depict the incidence of nausea and vomiting over a 3-day postoperative period for each of the three groups. This graphical representation allowed us to observe the detailed incidence and the trend of change over time. We observed a consistent decrease in the incidence of nausea and vomiting across all three groups during the three-day period following the surgery. In the NEPA group, the incidence of nausea and vomiting was initially low on the first day. On the second day, the incidence of vomiting reached zero, and the incidence of nausea approached zero as well. By the third day, neither nausea nor vomiting was reported in this group. Comparatively, the ONDA group exhibited a lower incidence of nausea and vomiting compared to the Control group throughout the three-day period. However, nausea and vomiting persisted until the third day, with the incidence of nausea still approximately 10%.

**Figure 2:**
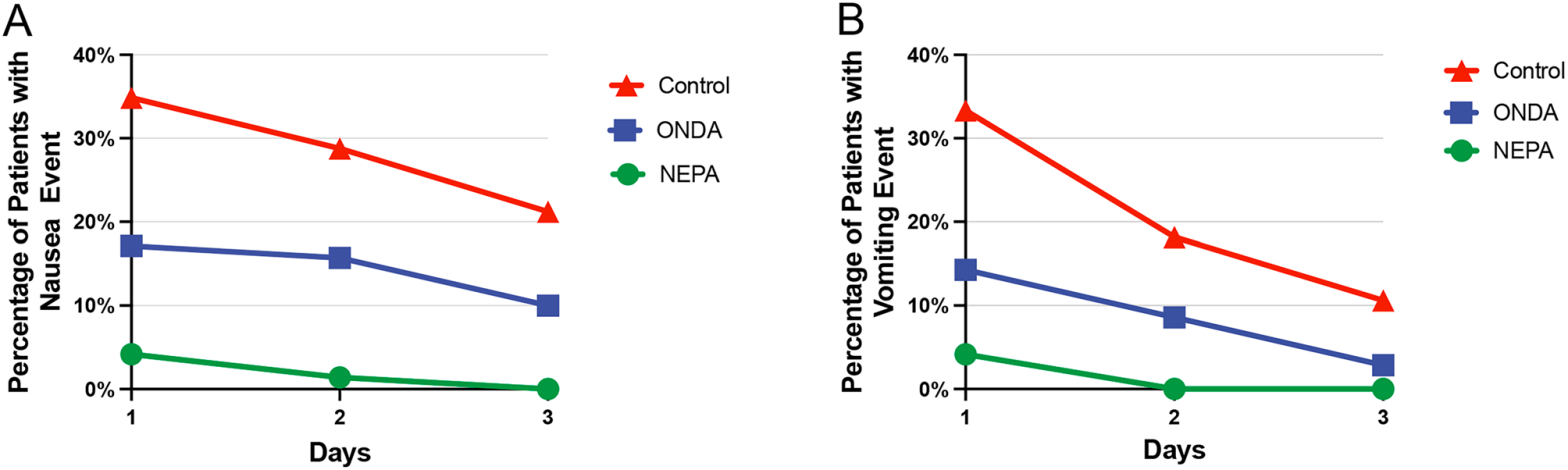
Line graph illustrating the incidence of nausea (**A**) and vomiting (**B**) across a 3 day postoperative period for each of the three distinct groups.

These findings provide evidence that the use of NEPA is effective in rapidly alleviating the occurrence of PONV in patients undergoing VATS radical resection of lung cancer (Fig. 2A,B).

### Comparison of the severity of PONV following VATS radical resection of lung cancer

During the data collection period, patients were asked to self-rate the severity of the symptoms based on the predefined criteria established for this study. The severity was stratified into four categories: absent, mild, moderate, and severe.

**Figure 3:**
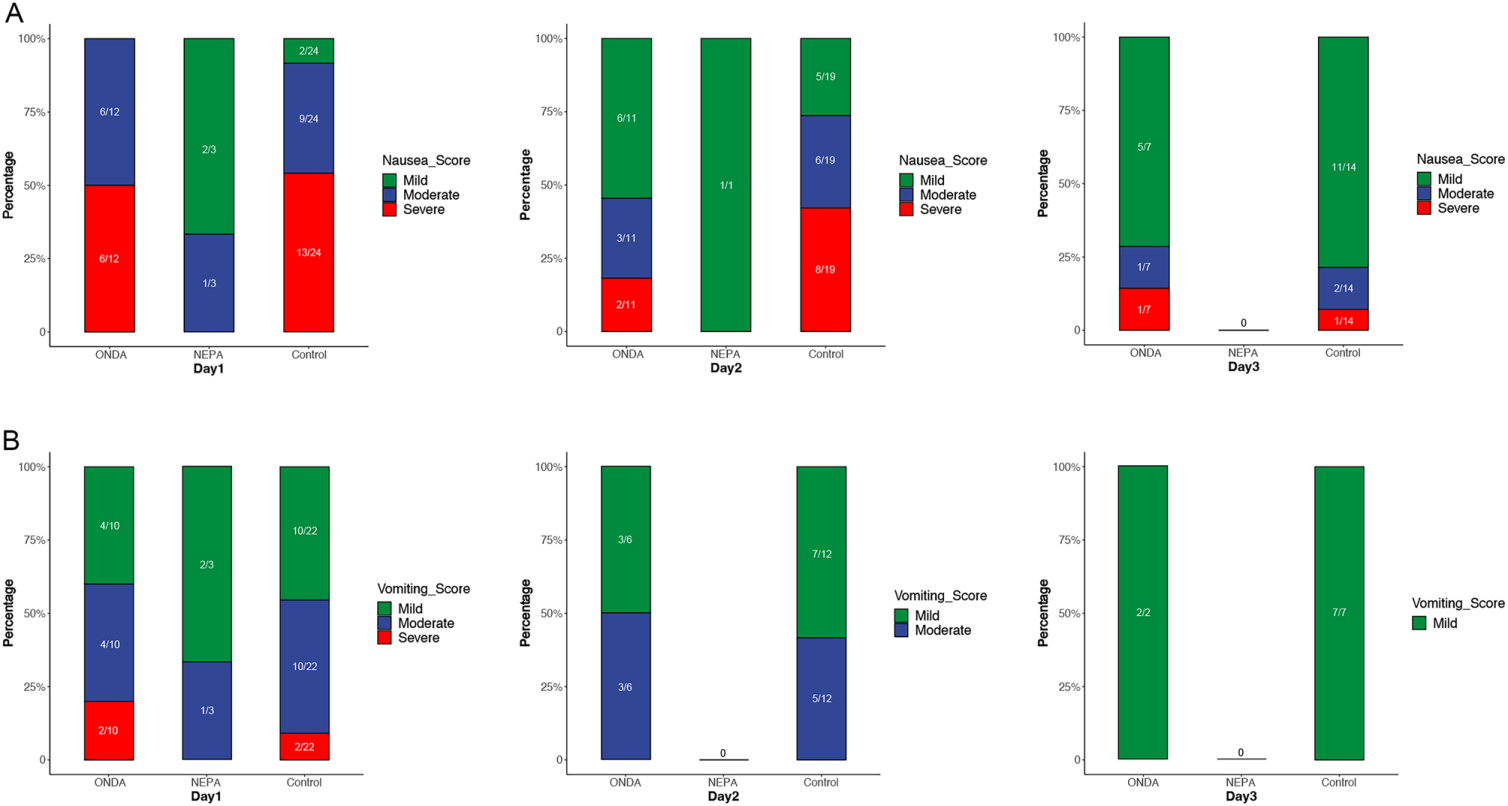
Analysis of severity of major adverse events following VATS radical resection of lung cancer across different groups. The stacked bar graphs illustrate the varying degrees of nausea (**A**) and vomiting (**B**) experienced by patients on the first, second, and third days following VATS radical resection of lung cancer. The severity of symptoms is color-coded: red bars indicate severe symptoms, blue bars denote moderate symptoms, and green bars represent mild symptoms. Each bar reflects the proportion of patients experiencing each severity level in relation to the total number of patients within different subgroups.

Initially, patients were stratified based on the severity of PONV at three days post-surgery. Those exhibiting either no symptoms or only mild symptoms were classified into the ‘Absent/Mild’ group, while patients with moderate to severe symptoms were allocated to the ‘Moderate/Severe’ group. A chi-square test was subsequently employed to evaluate the distribution differences among the NEPA, ONDA, and control groups, specifically examining the effectiveness in reducing the severity of nausea and vomiting across these groups. The analysis revealed statistically significant differences in both nausea severity (χ^2^ = 25.115, p = 3.519e–06) and vomiting severity (χ^2^ = 10.905, p = 0.004286) among the groups (Table S6). Subsequently, for a more detailed analysis of the difference in nausea and vomiting severity relief across the three groups, stacked bar graphs were employed. These graphs display the gradations in symptom severity experienced by patients on the first, second, and third postoperative days. Regarding nausea symptoms (Fig. 3A), in the NEPA group, the majority of cases in the NEPA group were classified as mild or moderate on the first day. In contrast, both the ONDA and Control groups had a higher proportion of severe cases, with only a small proportion experiencing mild symptoms. On the second day, all cases in the NEPA group had mild symptoms, while the proportion of severe cases in the ONDA and Control groups exhibited a decreasing trend, accompanied by an increase in the proportion of mild cases. By the third day, no cases of nausea were reported in the NEPA group, and the remaining cases in the ONDA and Control groups primarily exhibited mild symptoms. Furthermore, regarding vomiting symptoms (Fig. 3B), the severity across all three groups on the first day was mainly categorized as mild to moderate. Notably, no severe cases were reported in the NEPA group. From the second day postoperatively, the NEPA group was free of vomiting symptoms, while the ONDA and Control groups still had mild cases on the third day.

Based on these findings, it can be inferred that NEPA has the potential to alleviate the severity of PONV symptoms in patients following VATS radical resection of lung cancer.

### The protective effect of NEPA in preventing PONV following VATS radical resection of lung cancer

**Figure 4:**
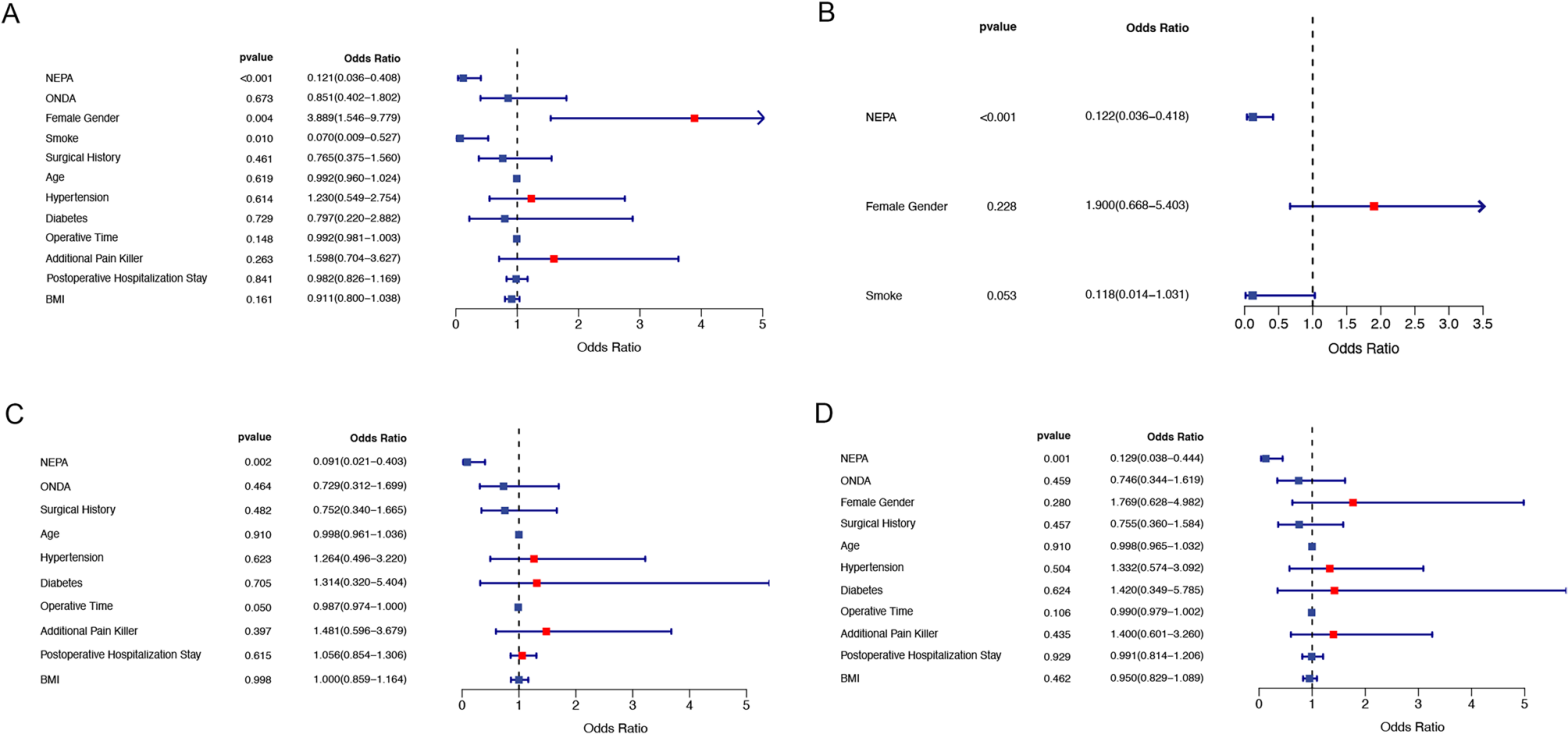
Investigation of the association between NEPA, clinical variables, and the occurrence of PONV in patients post-VATS radical resection of lung cancer conducted through univariate (**A**) and multivariate (**B**) logistic regression analyses. Examination of the correlation between NEPA use, clinical variables, and PONV incidence post-VATS radical resection of lung cancer within female (**C**) and non-smoking status (**D**) subgroups, utilizing univariate logistic regression analysis.

In our investigation, we employed the incidence of postoperative PONV as the study endpoint, integrating pertinent clinical variables including NEPA usage, ONDA usage and other factors into a logistic regression analysis. In the univariate logistic regression analysis, we determined that the use of NEPA and a smoking status were linked to a decreased risk of PONV, while being female was associated with an elevated risk. (P < 0.05, Fig. 4A). Subsequently, factors that demonstrated statistical significance in the univariate logistic regression analysis were selected for inclusion in the multivariate logistic regression analysis. The result of multivariate logistic regression analysis corroborated these findings, emphasizing the substantial impact of NEPA on the outcome after accounting for other variables (OR = 0.122, P < 0.001, Fig. 4B). Furthermore, in a univariate logistic analysis regression focusing on a subgroup of female and non-smoking patients, NEPA use was significantly correlated with PONV alleviation (P < 0.05, Fig. 4C,D).

## Discussion

In this study, our objective was to compare the efficacy of two antiemetic drugs in preventing perioperative PONV in patients undergoing VATS radical resection of lung cancer. The results of our study demonstrated that both NEPA and ONDA, when used prophylactically, significantly reduced the incidence of PONV in these patients. However, it was observed that NEPA exhibited superior efficacy in controlling the occurrence and severity of PONV compared to ONDA. Furthermore, the prophylactic use of NEPA also showed potential in reducing the incidence of dizziness, a common side effect in these patients. Importantly, our study identified that the prophylactic use of NEPA independently served as a protective factor against PONV.

Nausea and vomiting are commonly experienced side effects in cancer patients undergoing chemotherapy and surgery^24,25^. The incidence of PONV following general anesthesia, without specific preventive measures, is approximately 30% when inhalation anesthetics are used^26^. It is important to note that the rates of PONV can vary across different types of surgeries. For instance, even with routine antiemetic prophylaxis, up to 30% of patients may experience PONV after breast cancer surgery^27^. Similarly, in patients undergoing general anesthesia for lobectomy in lung cancer, the incidence of PONV is reported to be 29.6%^7^. In the case of laparoscopic bariatric surgery, the prevalence of PONV ranges from 44 to 90%^28^. Certain surgical procedures, such as cholecystectomy, laparoscopic surgery, gynecological surgery, and ear, nose, and throat (ENT) surgery, may increase the risk of PONV^6^. PONV is known to have detrimental effects on the patient's recovery process, including an increased risk of aspiration, airway compromise, and complications such as surgical suture incision dehiscence, pneumothorax, and esophageal rupture^29^. Furthermore, PONV is strongly associated with patient satisfaction during hospitalization, and its prevention has been shown to significantly improve patient satisfaction^30^. Therefore, it is crucial to effectively prevent and treat PONV in clinical practice. In this study, the incidence of nausea was found to be 4.17% and 17.14% in patients who received prophylactic treatment with NEPA and ONDA, respectively. Similarly, the incidence of vomiting was 4.17% and 15.71% in the respective treatment groups. Importantly, both drugs demonstrated significantly lower incidence rates compared to the blank control group. These findings suggest that both NEPA and ONDA are effective in alleviating the occurrence of PONV in patients undergoing VATS radical resection of lung cancer. Despite the lower incidence of PONV in the NEPA group compared to the ONDA group and the control group, some NEPA patients still experienced PONV within the first 24 h after surgery. This indicates that individual physiological or genetic factors may affect NEPA's effectiveness in preventing PONV.

Consensus guidelines for the management of PONV acknowledge various risk factors associated with its occurrence, such as being female, younger age, higher surgical risk, use of volatile anesthetics, history of PONV or motion sickness, non-smoking status, postoperative opioid use, and prolonged anesthesia^31^. Among untreated patients with multiple known risk factors, the incidence of PONV can be as high as 80%^26^. Surgery itself can indeed be considered a risk factor for PONV, particularly in cancer treatment. Procedures involving the upper respiratory, nasal, and gastrointestinal regions (such as the throat, mouth, esophagus, and stomach) can lead to swallowing of blood or bleeding in the gastrointestinal tract (GI), which can disrupt GI physiology and mechanics, thereby influencing the development of PONV^32^. The duration of surgery and exposure to anesthetics, particularly volatile inhalation anesthetics, also play a role in the risk of PONV. Prolonged surgical exposure to anesthetics, especially with a surgical duration exceeding 30 min, increases the likelihood of PONV^29^. A study has shown that the incidence of PONV in adult patients undergoing procedures lasting less than 30 min is around 2.8%, whereas it rises to 27.7% for procedures lasting over 3.0 h^33^. In the context of percutaneous hepatic artery chemoembolization, factors such as alkaline phosphatase, Barcelona Clinic Liver Cancer staging, allodynia, chemotherapeutic agents, iodine oil dosage, and preoperative analgesic use have been identified as risk factors for nausea and vomiting^34^. The findings of this study align with previous research, indicating that being female and non-smoking status are indeed considered risk factors for PONV in patients undergoing lobectomy. The logistic regression analysis conducted in this study supports these findings, demonstrating an association between PONV and female gender as well as non-smoking status in patients undergoing VATS radical resection of lung cancer. Furthermore, the study revealed that the prophylactic use of NEPA was identified as an independent protective factor against the development of PONV following VATS radical resection of lung cancer. This suggests that using NEPA as a preventive measure can help mitigate the occurrence of PONV in these patients. However, what puzzles us is that the efficacy of NEPA in female patients appeared to be less significant than anticipated. This could be attributed to a higher physiological predisposition to PONV in females, suggesting that higher doses or combination therapies may be required to achieve optimal prophylactic outcomes. In summary, the evidence from our study indicates that NEPA effectively reduces the risk of PONV following VATS radical resection of lung cancer. The benefits of NEPA are also pronounced in high-risk populations, including female patients and nonsmokers.

The pathophysiology of PONV is intricate and multifaceted, encompassing the intricate interplay of various neurotransmitters and receptors within the central nervous system (CNS) and GI^32^. Notable neurotransmitters implicated in PONV include serotonin (5-HT), dopamine, and substance P. These neurotransmitters exert their influence by binding to specific receptors, such as 5-HT3, D2, and NK-1 receptors, which play pivotal roles in initiating and propagating the vomiting reflex^35^. NEPA, with a half-life of 96 h, is a synergistic combination of the NK-1 receptor antagonist netupitant and the highly selective 5-HT3 receptor antagonist palonosetron. By concurrently targeting both the NK-1 and 5-HT3 receptors, NEPA provides a robust and comprehensive strategy for PONV management^36,37^. In contrast, ONDA, boasting a half-life of 3.5 h, is a commonly employed antiemetic in PONV management, functioning specifically as a selective 5-HT3 receptor antagonist^38,39^. It is considered the “gold standard” antiemetic for reducing the risk of PONV, and its routine postoperative intravenous administration of 4 mg has been established for PONV prevention^40^. Through its inhibition of serotonin binding to 5-HT3 receptors, primarily in the GI and CNS, ONDA disrupts the 5-HT3 receptor-mediated signaling pathway, thereby suppressing the vomiting reflex, and lowering the incidence of PONV^41^. In this study, it was found that the prophylactic use of NEPA was more effective than ONDA in reducing the incidence of PONV in patients undergoing VATS radical resection of lung cancer. The advantage of NEPA was evident from the first to the third postoperative day, which may be partly due to its long half-life. Additionally, NEPA demonstrated the potential to mitigate the severity of PONV, as patients using prophylactic NEPA experienced less PONV compared to those using ONDA. These findings suggest that a combination of an NK-1 receptor antagonist and a 5-HT3 receptor antagonist, such as NEPA, may be a more suitable regimen for PONV prevention in patients undergoing VATS radical resection of lung cancer.

In this study, the prophylactic use of NEPA has demonstrated significant advantages in preventing and treating PONV in patients undergoing VATS radical resection of lung cancer. A notable observation was the reduced average length of hospital stay in the NEPA group compared to the ONDA and Control groups. These findings hold important implications for patient care and clinical practice, as they can guide healthcare professionals in individualizing care plans and improving the quality of life for patients during the post-operative recovery period, potentially leading to faster recovery. Furthermore, these results can serve as a catalyst for future clinical investigations, aimed at further exploring the benefits and limitations of different types of antiemetic drugs in the prevention and treatment of PONV. By generating more robust evidence, these studies can contribute to the development of comprehensive guidelines for the use of antiemetic drugs, ensuring that patients receive optimal treatment and care.

Our study has revealed the potential of NEPA in reducing PONV in VATS radical resection of lung cancer. Although the cost of NEPA may be higher than traditional medications, its potential advantages in improving patient satisfaction and reducing additional medical interventions due to PONV may make it cost-effective overall^42^. Therefore, NEPA could be considered for inclusion in current treatment regimens. However, the introduction of NEPA should consider its availability in different regions and medical insurance coverage. Medical institutions should develop specific implementation strategies for introducing NEPA based on their own conditions and patient needs. In light of this, future research should further explore the cost-effectiveness of NEPA and its applicability in different medical settings.

However, it is important to acknowledge the limitations of this study. Firstly, the small sample size utilized in this study may have limited its statistical power, potentially leading to the inability to detect certain differences in the between-group comparisons. This limitation may reduce the credibility of the observed effects and potentially obscure the true advantages of different treatment compared to other treatments. A larger sample size would enhance the study's ability to detect significant effects. Secondly, the data collected for this study were obtained from a single medical center, which introduces the possibility of bias related to center-specific factors such as patient demographics, treatment protocols, surgical techniques, and nursing practices. This may limit the generalizability of our findings, as different centers may have different patient baseline characteristics and treatment responses. Consequently, the generalizability of the findings to other centers and diverse patient populations may be limited. Lastly, as a retrospective study, there is a potential for biases and confounders that could impact the accuracy and reliability of the findings. Although we have tried to control these biases through statistical methods, it is not possible to completely eliminate their impact. Prospective studies that can control for potential biases and confounders more effectively are needed to provide stronger evidence.

Based on the findings of this study, future research can explore the effectiveness of NEPA in preventing PONV in different surgical settings (such as abdominal surgery, gynecological surgery, and neurosurgery), compare its effects with other antiemetic strategies, and assess its long-term efficacy and safety. Research on specific high-risk populations such as women and non-smoking patients, as well as conducting cost-effectiveness analyses, will help to fully understand the value of NEPA and optimize its application in clinical practice.

## Conclusions

In conclusion, our study demonstrates that the use of NEPA significantly reduces the incidence and severity of PONV in patients undergoing VATS radical resection of lung cancer, compared to ONDA and no pharmacological intervention. Furthermore, this alleviating effect on PONV was also observed in patients at high risk for PONV, including female and non-smoking patients. Notably, the NEPA group also benefited from a shorter postoperative hospitalization stay highlighting the comprehensive benefits of NEPA in enhancing postoperative recovery. Lastly, our findings indicate that the use of NEPA serves as an independent protective factor against PONV in patients undergoing VATS radical resection of lung cancer. Based on these findings, we have reason to believe that incorporating NEPA into the postoperative management protocol for patients undergoing VATS radical resection of lung cancer could optimize recovery and reduce PONV-related complications. However, this still requires multicenter, prospective studies to further validate.

### Supplementary Information

Supplementary Table S1.

Supplementary Table S2.

Supplementary Table S3.

Supplementary Table S4.

Supplementary Table S5.

Supplementary Table S6.

## Data Availability

Datasets are included within the manuscript. Data and materials can be made available by the corresponding author upon reasonable request.
